# Tailoring calcium modification and imaging-guided strategies in complex bifurcation PCI

**DOI:** 10.1093/ehjcr/ytag281

**Published:** 2026-04-22

**Authors:** Giovanni Monizzi, Andrea Savastano, Edoardo Conte

**Affiliations:** Division of University Cardiology and Cardiac Imaging, Galeazzi-Sant’Ambrogio Hospital IRCCS, Via Cristina Belgioioso 173, Milan 20100, Italy; Division of University Cardiology and Cardiac Imaging, Galeazzi-Sant’Ambrogio Hospital IRCCS, Via Cristina Belgioioso 173, Milan 20100, Italy; Department of Health Sciences, University of Milan, Via Festa del Perdono 7, Milan 20100, Italy; Division of University Cardiology and Cardiac Imaging, Galeazzi-Sant’Ambrogio Hospital IRCCS, Via Cristina Belgioioso 173, Milan 20100, Italy

**This editorial refers to ‘Coronary bifurcation stenting: selection of technique, side-branch protection, and prognostic relevant side-branch’, by K. Çiloğlu *et al*. https://doi.org/10.1093/ehjcr/ytag255.**

In the paper ‘Intravascular ultrasound-guided tip-detection antegrade dissection and re-entry as a bailout strategy for calcification-induced side-branch occlusion during complex percutaneous coronary intervention: a case report’,^1^ Kawai *et al*. present an elegant bailout strategy to manage side-branch dissection. By using intravascular ultrasound (IVUS) to guide antegrade dissection re-entry (ADR) with a penetration wire, the authors successfully restored coronary flow. This case is highly relevant, and a more in-depth discussion could further enhance its educational value for interventional cardiologists who may encounter similar scenarios in the catheterization laboratory.

In their comments, Çiloğlu *et al*.^2^ addressed some comments to the operator, highlighting the choice for a two-stent strategy and the choice on the way authors decided to protect the side branch (SB). We would like to enrich these comments by taking into account other educational points that come out from this paper.

First, the choice of the initial strategy deserves consideration. Rotational atherectomy for debulking of a heavily calcified lesion is well established, particularly in the presence of tight main-vessel stenosis. However, it is also associated with an increased risk of SB occlusion.^3^ In the present case, given the narrow carina angle and the presence of calcification at the SB ostium, as described by the Bifurcation Academic Research Consortium, intravascular lithotripsy (IVL) might have represented a valuable alternative. This approach could have allowed safer SB protection with a jailed wire, potentially reducing the risk of dissection and occlusion.^4^ The Shockwave system is currently approved for the treatment of severely calcified lesions, and IVUS evidence of concentric calcification has been associated with higher procedural success, as demonstrated in the Disrupt CAD III trial.^5^ Therefore, the first key message is the importance of tailoring the calcium-modification strategy to the specific bifurcation anatomy in order to minimize SB compromise.

Second, the bailout technique itself warrants attention. Antegrade dissection re-entry, adapted here to treat abrupt vessel closure, is commonly employed in chronic total occlusion (CTO) percutaneaus coronary intervention (PCI) and is associated with high procedural success rates. Nevertheless, its routine use outside CTO settings may be questioned due to potential risks. In this context, IVUS guidance represents a particularly refined approach and is consistent with the most recent ‘Global Consensus Recommendations on Improving the Safety of CTO Intervention’.^6^ Indeed, uncontrolled dissection with high-penetration wires may result in vessel perforation, pericardial effusion, or propagation of the dissection plane. Real-time IVUS visualization of the wire tip allows for precise control of the dissection and facilitates safe re-entry into the true lumen of the SB.

A further noteworthy technical aspect is the use of a double-guide catheter setup, enabling simultaneous IVUS imaging and rewiring with a dual-lumen SASUKE catheter. This strategy is particularly advantageous, as ADR-dedicated devices (e.g. dual-lumen microcatheters or re-entry systems, such as the MANTA device) often require larger guiding catheters (7–8 French) due to their bulk. In the present case, the absence of disease in a large left main coronary artery allowed the use of two 6F guiding catheters, effectively separating imaging from procedural manoeuvres and thereby simplifying the intervention.

In conclusion, careful upfront selection of the interventional strategy is crucial to reduce complications in bifurcation PCI. When complications do occur, several bailout techniques are available; among these, the integration of intravascular imaging may significantly improve both procedural safety and clinical outcomes.

## Data Availability

No new data were generated or analysed in support of this research.

## Lead author biography

Dr Edoardo Conte acquired a solid academic and clinical background in the field of cardiovascular imaging, sport cardiology, and myocardial/pericardial disease. He works as a clinical cardiology at Galeazzi-Sant'Ambrogio Hospital IRCCS in Milan since 2022 following several years of work dedicated to advanced cardiovascular imaging at Centro Cardiologico Monzino IRCCS in Milan. He successfully obtained EACVI certification in transthoracic echocardiography, cardiac CT, and cardiac MRI. He is a Fellow of the European Society of Cardiology (FESC), a core member of EAPC Young Community and a member of the Myocardial and Pericardial Disease Working Group of the European Society of Cardiology. He is the Deputy Editor of the *EHJ-Case Report*. He was among the co-authors of more than 250 research papers (all published on indexed journal with impact factor).

## References

[1] Kawai K, Ashida K, Hasegawa K, Semba T, Tomishima Y. Intravascular ultrasound-guided tip-detection antegrade dissection and re-entry as a bailout strategy for calcification-induced side-branch occlusion during complex percutaneous coronary intervention: a case report. Eur Heart J Case Rep 2025;10:ytaf646. 41624009 10.1093/ehjcr/ytaf646 PMC12854407

[2] Çiloğlu K, Seter B, Güner A. Coronary bifurcation stenting: selection of technique, side-branch protection, and prognostic relevant side-branch. Eur Heart J Case Rep 2026. doi:10.1093/ehjcr/ytag255 PMC13165404 42130654

[3] Gallinoro E, Monizzi G, Sonck J, Candreva A, Mileva N, Nagumo S, et al Physiological and angiographic outcomes of PCI in calcified lesions after rotational atherectomy or intravascular lithotripsy. Int J Cardiol 2022;352:27–32. 35120947 10.1016/j.ijcard.2022.01.066

[4] Lunardi M, Louvard Y, Lefèvre T, Stankovic G, Burzotta F, Kassab GS, et al Definitions and standardized endpoints for treatment of coronary bifurcations. J Am Coll Cardiol 2022;80:63–88. 35597684 10.1016/j.jacc.2022.04.024

[5] Hill JM, Kereiakes DJ, Shlofmitz RA, Klein AJ, Riley RF, Price MJ, et al Intravascular lithotripsy for treatment of severely calcified coronary artery disease. J Am Coll Cardiol 2020;76:2635–2646. 33069849 10.1016/j.jacc.2020.09.603

[6] Wu EB, Kalyanasundaram A, Brilakis ES, Mashayekhi K, Tsuchikane E, Rafeh NA, et al Global consensus recommendations on improving the safety of chronic total occlusion interventions. Heart Lung Circ 2024;33:915–931. 38839467 10.1016/j.hlc.2023.11.030

